# Apoptosis and necrosis mediate skeletal muscle fiber loss in age‐induced mitochondrial enzymatic abnormalities

**DOI:** 10.1111/acel.12399

**Published:** 2015-09-14

**Authors:** Nashwa Cheema, Allen Herbst, Debbie McKenzie, Judd M. Aiken

**Affiliations:** ^1^ Department of Biological SciencesCentre for Prions and Protein Folding DiseasesUniversity of AlbertaEdmontonABCanada; ^2^ Department of AgriculturalFood and Nutritional SciencesCentre for Prions and Protein Folding DiseasesUniversity of AlbertaEdmontonABCanada

**Keywords:** aging, apoptosis, cell death, ETS abnormalities, mitochondria, necrosis, sarcopenia, skeletal muscle

## Abstract

Sarcopenia, the age‐induced loss of skeletal muscle mass and function, results from the contributions of both fiber atrophy and loss of myofibers. We have previously characterized sarcopenia in FBN rats, documenting age‐dependent declines in muscle mass and fiber number along with increased fiber atrophy and fibrosis in vastus lateralis and rectus femoris muscles. Concomitant with these sarcopenic changes is an increased abundance of mitochondrial DNA deletion mutations and electron transport chain (ETC) abnormalities. In this study, we used immunohistological and histochemical approaches to define cell death pathways involved in sarcopenia. Activation of muscle cell death pathways was age‐dependent with most apoptotic and necrotic muscle fibers exhibiting ETC abnormalities. Although activation of apoptosis was a prominent feature of electron transport abnormal muscle fibers, necrosis was predominant in atrophic and broken ETC‐abnormal fibers. These data suggest that mitochondrial dysfunction is a major contributor to the activation of cell death processes in aged muscle fibers. The link between ETC abnormalities, apoptosis, fiber atrophy, and necrosis supports the hypothesis that mitochondrial DNA deletion mutations are causal in myofiber loss. These studies suggest a progression of events beginning with the generation and accumulation of a mtDNA deletion mutation, the concomitant development of ETC abnormalities, a subsequent triggering of apoptotic and, ultimately, necrotic events resulting in muscle fiber atrophy, breakage, and fiber loss.

## Introduction

Sarcopenia, the involuntary loss of skeletal muscle mass and function with age (Rosenberg, 1997), contributes significantly to frailty and causes a decline in quality of life. In humans, 30% of the muscle mass is lost between 20 and 80 years of age (Frontera *et al*., 2000). The muscle mass decline observed in sarcopenia is due to fiber atrophy and loss (Lexell *et al*., 1986). There are several proposed mechanisms for the decline of muscle mass including altered satellite cell function (Gallegly *et al*., 2004), decrease in motor unit number (Roos *et al*., 1997), changes in hormone levels (Visser *et al*., 2003), accumulation of mitochondrial DNA deletions (Wanagat *et al*., 2001), and loss of myocytes via apoptosis (Dupont‐Versteegden, 2005).

Myofiber loss is an important component of sarcopenia, yet the cell death pathways involved in the process are not known. There are similarities in the apoptotic pathways in postmitotic multinucleated skeletal muscle and mononuclear mitotic cells, including cellular/nuclear shrinkage and activation of caspase‐3 (McArdle *et al*., 1999). In multinucleated myofibers, however, individual nuclei decay and cellular apoptosis occurs segmentally over an extended period of time (Dupont‐Versteegden, 2005). The mitochondrion is an important regulatory center for cell fate decisions as it sequesters factors that, upon release, lead to initiation of apoptosis. Mitochondrial‐mediated apoptosis involves the Bcl‐2 family members that mediate the release of cytochrome *c*, AIF, and Endo G into the cytosol as well as subsequent caspase activation. p53 upregulated modulator of apoptosis (PUMA) and Bid are key apoptotic factors of the Bcl‐2 family. Caspase‐8 cleaves Bid, generating tBid, which is then translocated to the mitochondria to promote the release of cytochrome *c* (Dirks & Leeuwenburgh, 2005). Similarly, PUMA is translocated to the mitochondria in response to p53 stress where it promotes apoptosis by inhibiting anti‐apoptotic proteins (Yee & Vousden, 2008).

Numerous lines of evidence suggest an involvement of apoptosis in myocyte loss. Downregulation of the apoptotic pathway can reduce the decline in muscle mass and function in aged animals (Dirks & Leeuwenburgh, 2004; Marzetti *et al*., 2009). Upregulation of the apoptotic pathway has been identified in premature aging models including mice lacking the antioxidant enzyme copper/zinc‐dependent superoxide dismutase (CuZnSOD or Sod1) that exhibit accelerated sarcopenia (Jang *et al*., 2010), as well as interleukin‐10‐deficient mice that exhibit extreme frailty (Walston *et al*., 2008). Increased levels of DNA laddering and caspase‐3 activity have been observed in transgenic mice expressing defective mitochondrial polymerase (Hiona *et al*., 2010).

Apoptosis occurs in muscle myonuclei as evidenced by the observation of DNA strand breaks and expression of pro‐apoptotic proteins Bax, caspase‐3, AIF and Apaf‐1, reviewed in Alway *et al*. (2011). In human muscle, increased levels of TUNEL‐positive nuclei have been detected from biopsies (Strasser *et al*., 1999; Malmgren *et al*., 2001; Whitman *et al*., 2005). An increase in caspase‐3 levels was not detected (Whitman *et al*., 2005). These studies are, however, all homogenate‐based with only one study, in 26‐month F344 rats (Dirks & Leeuwenburgh, 2004), detecting increased levels of cleaved caspase‐3, the active form of the effector caspase.

Cellular necrosis is a second significant mechanism of cell death. Prominent features of necrosis are ATP depletion, loss of ion homeostasis and membrane polarity leading to rapid swelling of the cell, membrane rupture, and subsequent release of cellular contents. Necrosis is neither organized nor executed in a similar manner to apoptosis, and cell death is a consequence of irreparable damage (Henriquez *et al*., 2008). Necrosis therefore frequently occurs during pathological conditions including stroke, ischemia, and neurodegenerative disorders (Syntichaki *et al*., 2002; Malhi *et al*., 2006; Henriquez *et al*., 2008). Inflammation is associated with necrosis and not with apoptotic cell death (Scaffidi *et al*., 2002). The complement system, a major component of innate immunity, responds to inflammation (Carroll, 2004). Activation of the complement cascade leads to assembly of terminal components and insertion of the C5b‐9 membrane attack complex (MAC) into the lipid bilayer of the plasma membrane. Assembly of MAC complexes results in the formation of transmembrane channels, loss of membrane integrity (Bhakdi & Tranum‐Jensen, 1978), leakage of intracellular contents, and macrophage infiltration to the site of inflammation (Kharraz *et al*., 2013).

There is considerable experimental evidence for a mitochondrial role in the fiber loss associated with sarcopenia. Skeletal muscle from aged rats (Wanagat *et al*., 2001), monkeys (Lee *et al*., 1993, 1994), and humans (Bua *et al*., 2006) displays an increase in the number of fibers exhibiting aberrations in the electron transport chain (ETC). These ETC abnormalities are visualized as loss of COX activity (COX−) as well as succinate dehydrogenase hyperactivity (SDH++) and are both focal (occur in individual cells) and segmental (occur within small regions of an individual cell) (Wanagat *et al*., 2001). Electron transport chain‐abnormal regions vary in length with the longer abnormal regions being more prone to fiber atrophy and breakage (Bua *et al*., 2004). Mitochondrial DNA deletion mutations are concomitant with ETC abnormalities (Lee *et al*., 1993, 1994; Müller‐Höcker *et al*., 1993; Johnston *et al*., 1995; Cao *et al*., 2001; Wanagat *et al*., 2001). Studies combining microdissection of individual fiber sections with quantitative PCR demonstrate that ETC‐abnormal fibers always have deletion mutation accumulations accompanying them (Cao *et al*., 2001; Wanagat *et al*., 2001; Gokey *et al*., 2004). Further studies showed a high abundance of mtDNA deletion mutations past a threshold value of > 90% resulting in the disruption of the ETC activity (Bua *et al*., 2006; Herbst *et al*., 2007). Together, these data confirm that mtDNA deletion mutations accumulate in individual muscle fibers, disrupting the ETC. This dysfunction of the ETC results in energy deficiencies, fiber atrophy and, ultimately, fiber loss.

To understand the role of apoptosis and necrosis in aged muscles, we used a histological approach to localize cell death events to individual muscle fibers. Five diverse markers of apoptosis and necrosis were examined, each having differential specificity to these processes. Bid truncation is an early event of apoptosis but does not necessarily dictate an apoptotic fate. PUMA, known to be transcriptionally activated in ETC‐abnormal fibers (Herbst *et al*., 2013), is a transcriptional response to p53 activation and influences Bid truncation by binding to Bcl‐2 and inducing membrane permeability transition pore formation. By contrast, activation of caspase‐3 is typically considered a committed step of apoptosis and directs proteolytic cleavage of cellular components. Two markers of necrosis were chosen encapsulating the loss of membrane polarity (C5b‐9) as well as the proinflammatory response of macrophages to the necrotized fiber (CD68). Each of these markers of cell death indicates different stimuli, responses, and progression which collectively help to define the cell fate decision and outcome.

By localizing the activation, execution, and characteristics of cell fate effector pathways to individual muscle fibers, we show that the predominant cause of cell death pathway activation in aged rat muscle is mitochondrial abnormalities. Mitochondrial abnormalities accumulate in an age‐dependent manner, initiate apoptotic and necrotic pathways as the dysfunctional region expands along the length of the myofiber and, thus, contribute to fiber atrophy and fiber loss in sarcopenia.

## Results

### Sarcopenic change in aged rats

As previously observed, there is a loss of muscle mass in the quadriceps of aged rats. In this study, 12‐month‐old rat quadriceps mass averaged 8.5 ± 0.15 g, while 36‐month‐old rat quadriceps weighed 4.6 ± 0.23 g. As expected, fiber loss was also evident with the rectus femoris of 36‐month‐old rats averaging 6484 ± 477 fibers compared to 12‐month‐old rats with an average of 9169 ± 447 fibers.

### Activation of cell death pathways is increased in the quadriceps of aged rats

**Figure 1:**
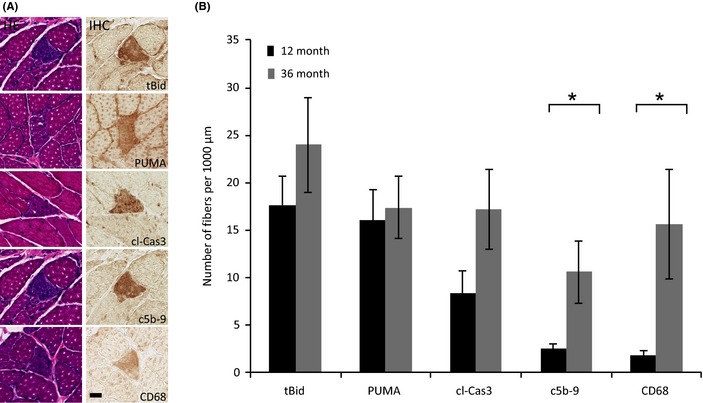
Detection of myofiber cell death in the quadriceps of 12‐ and 36‐month‐old rats. (A) Representative micrographs containing a fiber positive for cell death. The tissue sections were stained with hematoxylin and eosin or immunostained using antibodies against truncated form of Bid, PUMA, cleaved caspase‐3, C5b‐9, and CD68. The scale bar = 25 μm. (B) Number of fibers per 1000 μm of tissue analyzed that stained positive for the cell death markers in 12‐ and 36‐month‐old rat quadriceps. *Significant, *P* < 0.05, increase in aged rats. Data presented as mean ± SEM.

To determine the abundance of individual muscle fibers undergoing cell death, immunohistochemical analysis was performed on adult (12‐month‐old) and aged (36‐month‐old) rat quadriceps muscles for markers indicative of apoptosis [truncated form of Bid (tBid), PUMA, cleaved caspase‐3 (cl‐Cas3)] and necrosis (C5b‐9 and CD68) (Fig. 1A). Fibers that stained positive for any cell death marker at a 100‐μm interval were counted, annotated, and followed throughout the 1 mm of tissue. In the aged rectus femoris, there is a significant increase in the fraction of fibers positive for cl‐Cas3 (*P* = 0.03), C5b‐9 (*P* = 0.02), and CD68 (*P* = 0.03) (Table 1). In the 36‐month‐old rat quadriceps, there is a significant eightfold (*P* = 0.047) and ninefold (*P* = 0.048) increase in the number of fibers staining positive for C5b‐9 and CD68, respectively, compared to the 12‐month‐old rat. There is also a trend toward an increase in the numbers of myofibers positive for tBid and cl‐Cas3 with a threefold difference observed in aged rats (Fig. 1B).

**Table 1:** Abundance of fibers staining positive for cleaved caspase‐3, C5b‐9, and CD68 in 1000 μm of rectus femoris from 12‐ and 36‐month‐old rats

	Fibers staining positive		Percentage of fibers	
	Adult	Aged	Adult	Aged
cl‐Cas3	1.2 ± 0.4	5.6 ± 2.0	0.012 ± 0.004	0.08 ± 0.02^a^
C5b‐9	0.6 ± 0.2	2.4 ± 0.7^a^	0.006 ± 0.003	0.03 ± 0.02^a^
CD68	0.2 ± 0.2	2.0 ± 0.7^a^	0.002 ± 0.002	0.03 ± 0.01^a^
Fiber number in RF muscle	9169 ± 447	6484 ± 477^a^		

a Significant, *P* < 0.05, myofibers positive for cell death markers in aged rats (*n* = 5) and fiber loss (*n* = 4) in rectus femoris. Data presented as mean ± SEM.

### Cell death markers localize to ETC‐abnormal regions

**Figure 2:**
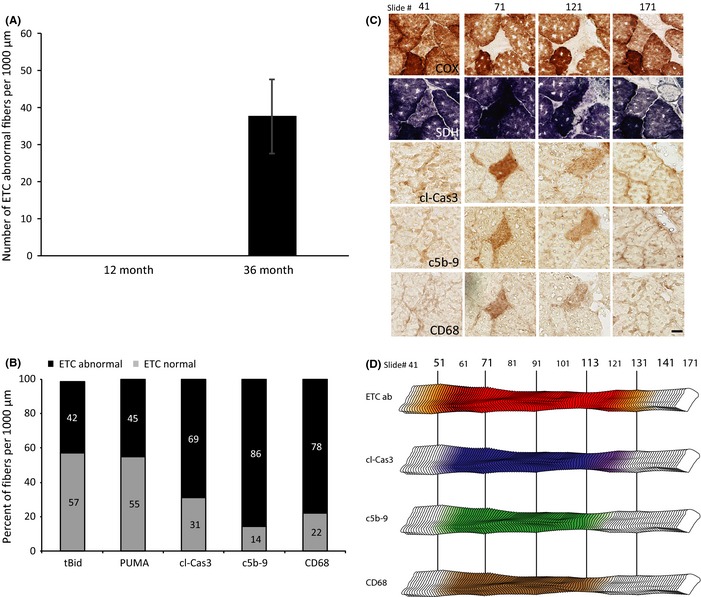
Myofiber cell death in electron transport chain (ETC)‐abnormal fibers of 36‐month‐old rats. (A) Abundance of ETC‐abnormal fibers per 1000 micrometers analyzed in the quadriceps of 12‐ and 36‐month‐old rats. No ETC‐abnormal fibers were detected in 12‐month‐old rats. Data presented as mean ± SEM. (B) The percent of ETC enzymatic phenotype of myofibers positive for apoptotic and necrotic markers in the rectus femoris of aged rats. (C) Representative ETC‐abnormal fiber undergoing cell death. Micrographs of histochemical staining for COX and SDH activity and immunostaining with antibodies to cleaved caspase‐3, C5b‐9, and CD68 are presented. The scale bar = 25 μm. (D) Digital reconstruction of the fiber in panel C. The red region indicates the ETC‐abnormal region, and the yellow region depicts transition area within the fiber. Increased staining for cleaved caspase‐3, C5b‐9, and CD68 is depicted in blue, green, and brown, respectively.

To ascertain the involvement of mitochondrial enzymatic abnormalities in age‐induced cell death processes, we stained tissue sections for COX and SDH activities. Mitochondrial enzyme activities were followed at 100‐μm intervals for 1000 μm to define the mitochondrial ETC phenotype. Within the 1 mm of 36‐month‐old rat quadriceps sectioned and analyzed, 39 ± 10 ETC‐abnormal (COX^−^/SDH^+++^ phenotype) fibers were identified per rat. No ETC‐abnormal fibers were observed in the 12‐month‐old rat quadriceps samples (Fig. 2A). We found a strong linkage between the regions of fibers positive for cell death markers and mitochondrial enzymatic abnormalities. Sixty percent of the fibers positive for any of the cell death markers were concomitant with ETC abnormalities. 42% of myofibers positive for tBid, 45% of myofibers positive for PUMA, 69% of myofibers positive for cl‐Cas3, 86% of myofibers positive for C5b‐9, and 78% myofibers positive for CD68 were ETC abnormal (Fig. 2B). A digital reconstruction of a representative COX^−^ and SDH^+++^ fiber from an aged rat illustrates the linkage between cell death pathway activation and the ETC‐abnormal region (Fig. 2C,D).

**Figure 3:**
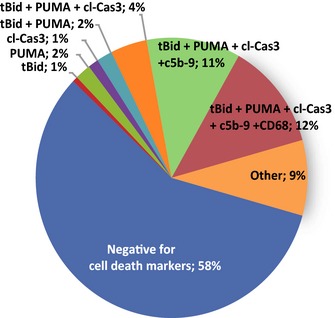
Prevalence of cell death in electron transport chain (ETC)‐abnormal fibers in 36‐month‐old rats. The percent of ETC‐abnormal fibers from quadriceps of 36‐month‐old rats that were positive for one or more cell death marker in the different combinations detected.

Approximately half (42%) of the ETC‐abnormal fibers were positive for apoptotic and/or necrotic markers. Ten percent of the ETC‐abnormal fibers were positive for only apoptotic markers. A majority of these fibers immunostained positive for all three apoptotic markers (tBid, PUMA, and cl‐Cas3). Thirty‐two percent of the ETC‐abnormal fibers were positive for both apoptosis and necrosis with 37% staining positive for all five markers of apoptosis and necrosis (Fig. 3).

### ETC abnormality length is positively correlated with cell death pathway activation

**Figure 4:**
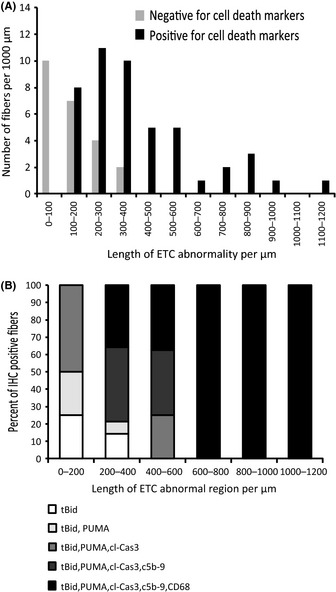
Fibers with longer electron transport chain (ETC)‐abnormal regions are positive for cell death markers. (A) A significant increase is observed in ETC‐abnormal length for fibers positive in cell death markers, *P* value < 0.0001 (*n* = 68 fibers). (B) The length of ETC‐abnormal region was measured for fibers positive for only tBid and subsequent addition of PUMA, cl‐Cas3, C5b‐9, and CD68. Fibers with the longest ETC‐abnormal region are positive for all five cell death markers. A total of 32 fibers were analyzed.

Our histological approach, the sectioning of one hundred consecutive 10‐μm sections, permitted measurement of ETC‐abnormal region length. We previously demonstrated that the length of ETC‐abnormal regions in an aged muscle fiber varies, with fibers containing longer ETC abnormalities being more prone to atrophy and fiber breakage (Bua *et al*., 2004). Fibers positive for cell death markers have significantly longer ETC abnormality lengths with a range of 200–1200 μm, whereas fibers that did not immunostain with any cell death marker have shorter ETC abnormalities (range of 100–400 μm, Fig. 4A). Myofibers positive for tBid exclusively and fibers positive for both tBid and PUMA had the shortest length of ETC‐abnormal region. The range of ETC abnormality length for tBid‐positive fibers was 200–300 μm. tBid‐ and PUMA‐positive fibers had an ETC abnormality length of 200–400 μm. Fibers positive for all three apoptotic markers, tBid, PUMA, and cl‐Cas3, had an ETC abnormality length of 200–500 μm. Fibers staining for tBid, PUMA, cl‐Cas3, and C5b‐9 had an ETC abnormality length of 300–600 μm. Electron transport chain‐abnormal fibers positive for all apoptotic and necrotic markers (tBid, PUMA, cl‐Cas3, C5b‐9, and CD68) had the longest ETC‐abnormal regions. One hundred percent of the ETC‐abnormal fibers (*n* = 6 fibers) with an abnormality length of > 600 μm were positive for all cell death markers (Fig. 4B).

### ETC abnormality intrafiber atrophy is positively correlated with cell death pathway activation

**Figure 5:**
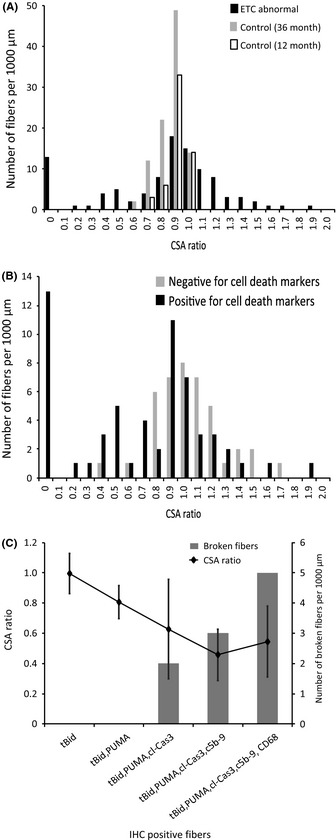
Apoptosis and necrosis are prevalent in atrophic and broken electron transport chain (ETC)‐abnormal fibers. (A) Intrafiber atrophy is more prevalent in ETC‐abnormal fibers (*n* = 102 fibers) than in ETC‐normal fibers from either 36‐month‐old (*n* = 99 fibers) or 12‐month‐old (*n* = 30 fibers) rats. Significant variance (*P* value < 0.0001) in cross‐sectional area ratio (CSAR) values between ETC‐abnormal and ETC–normal fibers. (B) There is increased atrophy in ETC‐abnormal fibers positive for cell death. Cross‐sectional area ratios of COX−/SDH++ fibers and COX‐normal/SDH‐normal fibers were determined. Significant atrophy in ETC‐abnormal fibers (*P* value = 0.0016). (C) Atrophic and broken ETC‐abnormal fibers stain positive for cleaved caspase‐3, C5b‐9, and CD68. A total of 32 fibers were analyzed.

The cross‐sectional area ratios (CSARs) of individual fibers were calculated and graphed to generate an intrafiber atrophy profile. A CSAR value of 0 indicates fiber breakage and a value ≥ 1 indicates an absence of atrophy (Bua *et al*., 2002). Significantly less atrophy was observed in ETC‐normal fibers of 12‐ and 36‐month‐old rats than in ETC‐abnormal fibers of 36‐month‐old rats (Fig. 5A). Electron transport chain‐abnormal fibers positive for cell death markers have a significant increase (*P* = 0.0016) in intrafiber atrophy with an average CSAR of 0.6 compared to ETC‐abnormal fibers that are negative for cell death, which have an average CSAR of 1 (Fig. 5B). Atrophic and broken ETC‐abnormal fibers were consistently positive for apoptosis and necrosis, indicating that cell death pathway activation mediates fiber breakage and loss.

**Figure 6:**
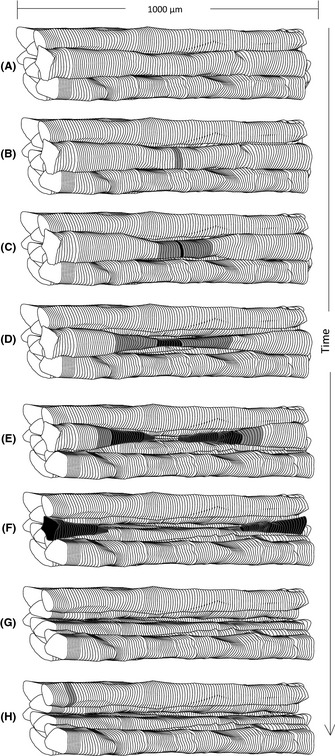
Model of myofiber loss. (A) Bundle of hypothetical fibers that contain wild‐type mtDNA with normal electron transport chain (ETC) function. (B) A mtDNA deletion mutation, presumably resulting from an mtDNA replication error, is generated. The deletion‐containing mtDNA genomes accumulate in a segment of fiber disrupting ETC enzymatic activity (gray). (C) The deficiency results in activation of Bid and PUMA (black). (D) As the ETC‐abnormal region expands, apoptosis is mediated by cl‐Cas3 leading to intrafiber atrophy. (E) Upon the activation of apoptosis and necrosis, fiber breakage occurs within the ETC‐abnormal region. (F) Apoptotic and necrotic region expands in the fiber. (G) Fiber loss occurs. (H) Another individual fiber, with accumulation of a deleted mtDNA genome, undergoes C–G again.

Fibers positive for cl‐Cas3, C5b‐9, and CD68 exhibited more intrafiber atrophy. The average CSAR of fibers positive for tBid, PUMA, and cl‐Cas3 was 0.627 ± 0.329. If C5b‐9 is involved, the average CSAR was 0.457 ± 0.169, and with CD68, the average CSAR was 0.544 ± 0.234. Atrophy was not evident in fibers positive exclusively for tBid or tBid and PUMA. Furthermore, fiber breakage events within the ETC‐abnormal region were not detected when atrophy was not present (Fig. 5C). All broken fibers were positive for cl‐Cas3, C5b‐9, and/or CD68, suggesting that these markers were involved in the terminal stage of ETC‐abnormal fibers (Fig. 6).

## Discussion

The decline in fiber number in the aged rat cohorts used in this study is consistent with previous observations (Wanagat *et al*., 2001; Bua *et al*., 2008). Numerous studies document increased cell death in aged skeletal muscle (Dirks & Leeuwenburgh, 2004; Marzetti *et al*., 2009), reviewed by Alway (Alway *et al*., 2011). Electron transport chain abnormalities also accumulate with age and are concomitant with fiber atrophy and fiber breakage (Bua *et al*., 2002, 2004). However, the linkage between cell death processes and ETC‐abnormal fiber had not previously been explored.

### Mitochondrial‐mediated apoptosis

Mitochondrial function declines with advancing age (Bratic & Larsson, 2013). Stress stimuli induce the release of pro‐apoptotic factors sequestered in the mitochondria activating apoptotic signaling. With age, there is evidence suggesting that mitochondrial dysfunction activates apoptosis contributing to sarcopenia (Dirks & Leeuwenburgh, 2002; Pistilli *et al*., 2006; Chabi *et al*., 2008). Mitochondrial pro‐apoptotic proteins Bax, caspase‐3, AIF, and Apaf‐1 have been detected in aged and atrophying muscles (Dirks & Leeuwenburgh, 2004; Siu *et al*., 2005; Pistilli *et al*., 2006; Alway *et al*., 2011). Building upon the studies of Dirks and Leeuwenburgh, our histological approach allows us to identify the fibers and regions of fibers that were apoptotic. We determined that the predominant source of apoptotic stimuli is ETC‐abnormal skeletal muscle fibers.

### Apoptosis and necrosis in aged skeletal muscle fibers

Using a cell‐by‐cell scanning approach, we detected a significant increase in the number of fibers positive for cl‐Cas3 and necrosis markers within a subset of individual muscle fibers in aged rat quadriceps. Although previous studies have looked for activation of apoptosis in aged skeletal muscle, altered caspase activity has not been demonstrated with age (Dirks & Leeuwenburgh, 2004; Alway *et al*., 2011). These studies were homogenate‐based and, therefore, unable to discriminate between focal activation of apoptosis in a subset of muscle fibers vs. a more generalized pro‐apoptotic environment.

Necrotic fibers activate the complement system to assemble the C5b‐9 membrane attack complex. This assembly of complement components occurs primarily in early stages of necrosis (Gaipl *et al*., 2001) and allows for macrophage infiltration into the damaged fiber. We observed a significant increase in the number of fibers positive for necrosis markers (C5b‐9 and CD68) in aged skeletal muscle. Fibers with ETC abnormalities were especially prone to necrosis. Our data suggest that cleaved caspase‐3‐, C5b‐9‐, and CD68‐positive fibers are prevalent in the rectus femoris of aged rats and contribute to the age‐dependent loss of myofibers. Furthermore, ETC‐abnormal fibers that are atrophic exhibit a significant increase for apoptotic and necrotic markers in the abnormal region.

### Progression of cell death in aged muscle fibers

Both apoptotic and necrotic cell death markers exhibited an age‐dependent accumulation and segmental distribution in rat skeletal muscle. The majority of cell death marker‐positive regions were also concomitant with mitochondrial enzymatic abnormalities. This finding was unexpected because sarcopenia is generally considered to be multifactorial. This observation led us to characterize cell death processes in ETC‐abnormal fibers, and we determined that 42% of ETC‐abnormal fibers in aged quadriceps were positive for cell death markers. The length of ETC abnormalities varied from fiber to fiber. In general, fibers with small regions of ETC abnormalities were negative for markers of cell death. Longer ETC‐abnormal regions were more prone to fiber atrophy and exhibited high levels of staining for cell death markers. Furthermore, fibers positive for both apoptosis and necrosis markers are more likely to be ETC abnormal, providing strong evidence that a majority of the cell death events are triggered by ETC abnormalities. Fiber breakage was only observed in fibers undergoing both apoptosis and necrosis. Our data indicate that fiber loss observed in sarcopenia is due to the accumulation of ETC abnormalities and subsequent activation of apoptotic and necrotic pathways.

There is a correlation between the length of the ETC abnormality and cell death marker. For example, ETC‐abnormal fibers that did not stain positive for cell death markers tended to be of short length (100–400 μm), regions positive for tBid (while not staining for other cell death markers) were < 300 μm, and fibers positive for tBid and PUMA were < 400 μm. Fibers positive for tBid, PUMA, and cl‐Cas3 had a maximum length of 500 μm, with tBid‐, PUMA‐, cl‐Cas3‐, and C5b‐9‐positive fibers at 600 μm, while fibers positive for all five cell death markers were present in the longest ETC‐abnormal region, 1200 μm. These studies suggest a progression of cell death marker activation in ETC‐abnormal fibers with ETC‐abnormal regions initially not linked to cell death markers; as the ETC abnormality progressively expands, apoptosis markers (tBid, PUMA, and cleaved caspase‐3) are observed; and finally, with the longer ETC abnormalities, necrosis markers (C5b‐9 and CD68) are observed.

### Proposed model of fiber loss

We previously proposed a model of muscle fiber loss with age. The process begins with the generation and subsequent clonal accumulation of a mitochondrial DNA deletion mutation within an affected muscle fiber. The accumulation of deletion mutation‐containing mtDNA genomes results in the loss of cytochrome *c* oxidase activity and the generation of numerous cellular responses. In this manuscript, we demonstrate that both apoptotic and necrotic responses occur with necrosis being tightly linked to fiber atrophy/fiber breakage. In the regions of myofibers with mitochondrial dysfunction, activation of apoptosis is followed by necrosis. All breakage events occur within the ETC‐abnormal region and are positive for necrosis markers, suggesting that necrosis is responsible for fiber loss (Fig. 6). We detected ~70–80% of the total apoptotic and necrotic fibers in aged rat quadriceps to be ETC abnormal further supporting the hypothesis that mtDNA deletions and the resultant mitochondrial enzymatic abnormalities play a causal role in the etiology of sarcopenia.

## Methods

### Tissue preparation

Adult 12‐month (*n* = 5)‐ and aged 36‐month (*n* = 5)‐old male Fischer 344 × Brown Norway F1 hybrid rats were purchased from the National Institute on Aging colony maintained by Harlan Sprague Dawley (Indianapolis, IN, USA). Animals were euthanized, and the quadriceps muscles were dissected from the animals, bisected at the midbelly, embedded in optimal cutting temperature compound (Sakura Finetek, Torrance, CA, USA), flash‐frozen in liquid nitrogen, and stored at −80 °C. A minimum of one hundred 10‐μm‐thick consecutive transverse cross sections were cut with a cryostat at −20 °C and placed on probe‐on‐plus slides. Slides were stored at −80 °C until needed.

### Immunohistochemistry and histochemistry

At 100‐μm intervals, the fifth, sixth, seventh, eighth, and ninth tissue slides were fixed overnight in 10% buffered formalin. Antigens were retrieved by boiling in 10 mm citrate buffer, pH 6.0. Slides were blocked in TBS‐T containing 5% goat serum. Slides were incubated with primary antibodies in blocking solution overnight, followed by TBS‐T washes. Biotinylated goat anti‐rabbit IgG (1:200) and avidin–peroxidase enzyme complex (1:50) from VECTASTAIN^®^ ABC Kit were used according to the manufacturer's specification (Vector Laboratories, Burlingame, CA, USA). DAB was used for chromogenic detection. Primary antibodies used were rabbit anti‐activated caspase‐3 1:200 (Promega, Madison, WI, USA), rabbit anti‐PUMA 1:200 (Abcam, Cambridge, MA, USA), rabbit anti‐truncated Bid 1:200 (EMD Chemicals Inc., San Diego, CA, USA), rabbit anti‐C5b‐9 1:500 (Abcam), and rabbit anti‐CD68 1:1000 (Abcam). At 100‐μm intervals, the first tissue slide was stained for COX and the second for SDH as previously described (Wanagat *et al*., 2001). The third slide was dual‐stained, first for COX and secondly for SDH. Two slides within the series were used for hematoxylin and eosin (H&E) staining. After histochemistry or immunohistochemistry, the slides were digitally scanned in the microscope (NanoZoomer 2.0‐RS, Hamamatsu, Bridgewater, NJ, USA). Individual muscle fibers were followed throughout the 1 mm of tissue. Fibers positive for cell death markers and ETC abnormalities (COX^−^/SDH^+++^) were identified and annotated.

### Fiber counts of muscle cross section

The quadriceps muscle is composed of four major muscles, vastus intermedialis (VI), vastus medialis (VM), vastus lateralis (VL), and rectus femoris (RF). The RF muscle is completely surrounded by the vastus muscles, facilitating a dissection of an intact muscle. Therefore, the RF was used for fiber counts as the fiber number is more accurately and precisely determined compared to the whole quadriceps muscle. Individual muscle fibers were annotated on digital images of the cross section of rectus femoris muscle at the midbelly, using Adobe Photoshop (Adobe Systems, Inc., San Jose, CA, USA).

### Measurements of cross‐sectional area ratio and ETC abnormality length

Cross‐sectional area (CSA) of randomly selected individual ETC‐normal and ETC‐abnormal muscle fibers was measured throughout the 1000 μm using the nanozoomer digital pathology imaging software (Hamamatsu, Bridgewater, NJ, USA). The minimum CSA value in the ETC‐abnormal region was divided by the average value of the fiber CSA in the ETC‐normal region within the same fiber. In ETC‐normal fibers, the ratio between minimum CSA and average CSA was determined. The CSA ratios of ETC‐abnormal and ETC‐normal fibers were plotted on a histogram. The length of ETC abnormality was determined by counting the number of slides the abnormality appeared in and multiplying by 10 μm. Fibers that were ETC abnormal at the 100th slide were followed beyond 1000 μm until the fibers turned normal. Fibers that immunostained for each of the markers were annotated and followed throughout the 1000 μm.

### Statistical analysis

Data were presented as mean ± SEM for five aged and adult rats. Three aged rats were used when analyzing length and atrophy of ETC‐abnormal fibers. All statistical analysis was performed using graphpad prism version 5.00 for Windows (GraphPad Software, San Diego, CA, USA). Student's *t*‐tests were performed for all analysis with a *P* value < 0.05 being significant. A Welch's *t*‐test was performed on analysis of length of ETC abnormality to correct for unequal variance between the two data sets (Fig. 4A). To measure significant variance (Fig. 5A,B), *F*‐tests were performed for fiber atrophy analysis. To calculate percent of incidences, 125 immunopositive fibers were analyzed in Fig. 2B and 161 ETC‐abnormal fibers were analyzed in Fig. 3.

## Author contributions

The experiments were designed by NC, AH, DM, and JMA. All experiments were performed by NC. The manuscript was prepared by NC and edited by AH, DM, and JMA.

## Funding

Research was supported by NIH grant # R01 AG030423 to JMA.

## Conflict of interest

The authors declare no conflict of interest.

## Supporting information


**Table S1** Antibodies used for cell death analysis.
