# Supplementary material for: Apoptosis and necrosis mediate skeletal muscle fiber loss in age‐induced mitochondrial enzymatic abnormalities
Source: Aging Cell. 2015 Sep 14;14(6):1085–93. doi: 10.1111/acel.12399 (PMC4693455; doi:10.1111/acel.12399)
Supplement: Supplementary file 1 — Table S1 Antibodies used for cell death analysis. [file ACEL-14-1085-s001.docx]

**Supplementary Table 1**. Cell death markers.

| **Antibodies** | **Antigen** | **Cell death**  **pathway** | **Description** |
| --- | --- | --- | --- |
| **tBid** | Truncated form of Bid | Apoptosis | The cleavage of Bid is an initiating  step for apoptosis |
| **PUMA** | Bcl-2 family member that translocates to the  mitochondria | Apoptosis | PUMA initiates apoptosis by antagonizing  Bcl-X_L_ and promoting Bax multimerization  and mitochondrial translocation. |
| **Cleaved Caspase3** | Activated form of caspase 3 | Apoptosis | The effector caspase that activates CAD,  caspase activated DNAse, which cleaves  DNA into small fragments. |
| **C5b-9** | Membrane attack complex | Necrosis | *MAC* is composed of C5b to C9 (*C5b*-*9*) complement proteins and forms a pore on the necrotic cell. |
| **CD68** | Glycoprotein present on lysosomal membranes of macrophages | Necrosis | Indicates macrophage invasion allowing for phagocytosis of cellular debris and release of proinflammatory cytokines. |
